# Informal science, technology, engineering and math learning conditions to increase parent involvement with young children experiencing poverty

**DOI:** 10.3389/fpsyg.2022.1015590

**Published:** 2022-11-01

**Authors:** Tricia A. Zucker, Gloria Yeomans Maldonado, Michael Assel, Cheryl McCallum, Cindy Elias, John M. Swint, Lincy Lal

**Affiliations:** ^1^University of Texas Health Science Center at Houston, Houston, TX, United States; ^2^Children's Museum Houston, Houston, TX, United States; ^3^Department of Management, Policy and Community Health at University of Texas, School of Public Health University of Texas Health Science Center-Houston, Houston, TX, United States

**Keywords:** parent involvement, informal STEM, pre-kindergarten, family engagement, museum

## Abstract

Broadening participation in early science, technology, engineering and math (STEM) learning outside of school is important for families experiencing poverty. We evaluated variations of the Teaching Together STEM pre-kindergarten program for increasing parent involvement in STEM learning. This informal STEM, family engagement program was offered in 20 schools where 92% of students received free/reduced lunch. The core treatment included a series of family education workshops, text messages, and family museum passes. The workshops were delivered at school sites by museum outreach educators. We randomly assigned schools to business-as-usual control or one of three additive treatment groups. Using an additive treatment design, we provided the core program in Treatment A, we added take-home STEM materials in Treatment B, and added materials + parent monetary rewards in Treatment C. The primary outcome was parent involvement in STEM (*n* = 123). There were no significant impacts of any treatment on parent involvement; however, the groups that added take-home materials had larger effect sizes on parent involvement at posttest (ES = −0.08 to 0.18) and later, kindergarten follow-up (ES = −0.01 to 0.34). Adding parent monetary rewards only produced short-term improvements in parent involvement that faded at follow-up. We discuss implications for other community-sponsored family engagement programs focused on informal STEM learning, including considering characteristics of families who were more versus less likely to attend. These null findings suggest that alternatives to in-person family education workshops should be considered when parents are experiencing poverty and have competing demands on their time.

## Introduction

Students experiencing poverty have fewer opportunities for informal science, technology, engineering and math (STEM) during outside of school time (National Research Council, 2009). Although decades of disseminating home literacy research findings has given parents the clear message that reading at home is important, parents are less likely to have clear understanding that early math and science are important home learning activities (LeFevre et al., 2009; McClure et al., 2017). Nationally representative, U.S. datasets show that about 45% of parents read to their young child every day, but only about 12% talk about nature or science daily (Barnett et al., 2020). Yet families can readily support early, informal STEM in already-existing family activities that include STEM, such as cooking, grocery shopping, outdoor play, and games (McClure et al., 2017; Pattison et al., 2020). Decades of empirical evidence shows that parental involvement in learning is related to children’s academic achievement (Castro et al., 2015; Ma et al., 2016). Parents are more likely to get involved in their child’s learning in preschool than later grades (Welsh et al., 2020), making this an important period for family engagement programs. Yet optimal, effective methods for increasing parent involvement are not well understood. One meta-analysis showed widely-used approaches have little to no effect on long-term outcomes (Grindal et al., 2016). Parent involvement interventions may not be of sufficient intensity for families of lower socio-economic status (Puma et al., 2010) and school-based family education events may be hard for families experiencing poverty to attend (Marti et al., 2018; Barnett et al., 2020).

Therefore, this study examined three variations of a pre-kindergarten (pre-k) program called *Teaching Together (TT) STEM*, designed to increase parent involvement in science and math when delivered at schools where most families were experiencing poverty. The core component was family education workshops, an approach to family engagement that is widely-used in United States schools (National Center for Education Statistics, 2021). But given that workshops may be insufficient to change outcomes (Grindal et al., 2016), we compared this basic treatment to two randomly assigned levels of support that were designed to reduce barriers to parent involvement. Specifically, we added *materials* to a second treatment group with a set of bilingual, take-home STEM activity kits. We added these materials plus *monetary rewards* to a third treatment to reinforce parent involvement. This resulted in three randomly assigned treatment conditions and a control/business-as-usual (BAU) group.

### Support for *TT STEM* treatment conditions

The core *Treatment A* sought to increase parents’ knowledge and skills to facilitate home-based, informal STEM activities with their preschooler. This included up to six family education workshops hosted at participating schools in the library or cafeteria. At each workshop, bilingual (English/Spanish) outreach educators from a children’s museum modeled how to incorporate STEM during every day routines. These afterschools, museum-led workshops used a strengths-based approach that promoted playful, conversation-focused approaches to supporting science and math at home. At the start of workshops, families had pizza and met the museum facilitator. The museum educator explained that STEM is everywhere, showed a video of racially/ethnically diverse parents and children doing STEM, and modeled how to talk to your child about science and math during book reading. Then, families practiced supporting their child’s learning at five activity stations while the educator provided support and feedback. The approach focused on talking about science and math during already-existing activities in most families, such as cooking, shopping, and fixing things. At the end of workshops, families received free family admission passes to Children’s Museum Houston, as museum spaces uniquely spark early STEM interest (e.g., Haden, 2010). Parents also received a series of text message tips about counting, observing, comparing or other ways to integrate STEM into every day, playful activities. Texts are a low-cost nudge and effective support when they included actionable information for parents to support learning (Caspe and Lopez, 2018; Cabell et al., 2019). All written materials were bilingual and museum educators used a bilingual facilitation in schools serving a majority of bilingual families. These treatments were similar to other culturally-relevant family engagement approaches by using inclusive and strengths-based approaches (Puma et al., 2010), but were offered at schools rather than in other community spaces that may be more welcoming to some families (McWayne et al., 2022). Also, the treatments did not feature adaptations specific to racial/ethnic cultures (*cf.* Leyva et al., 2022).

The second *Treatment B* added nine take-home STEM kits because families experiencing poverty may have limited access to STEM-related materials and informational children’s books to facilitate learning (Reinhart et al., 2016; Neuman, 2017). Effective programs for supporting STEM knowledge often include family activity kits to support STEM inquiry at home (Clements and Sarama, 2008; Kaderavek et al., 2020). Meta-analytic reports conclude that both increasing parent involvement in learning and providing age-appropriate home learning materials are linked to children’s academic outcomes (Boonk et al., 2018). Increasing home learning resources may be particularly important for students who begin pre-k with limited math skills (Powell et al., 2012). *TT STEM* take-home STEM activity kits included inquiry-based activities with step-by-step photos, bilingual instructions, and aligned informational tradebooks.

The third *Treatment C* added rewards to motivate parents. It is possible that some parents require more than just information and materials to overcome negative cultural stereotypes or past experiences with science or math (McClure et al., 2017). Therefore, the third treatment added parent rewards of $2.50 per STEM activity completed. These extrinsic, monetary incentives were designed to demonstrate the value of doing STEM with your child while also offsetting potential perceived costs, such as effort demands or lost time for alternative activities (Parker et al., 2017). Other experimental studies with parents of preschoolers show rewards of $0.50 for completing book reading sessions effectively increase parent involvement (Justice et al., 2018). Although the argument against monetary rewards in parenting programs is that they are unlikely to feasible in practice when offering no-cost family engagement programs, some experimental evidence shows monetary incentives increase the proportions of low-income families that complete parenting interventions (e.g., Heinrichs, 2006). Yet, other experiments show limited value of monetary incentives (Dumas et al., 2010). Thus, this variable warrants further study.

### Study purpose

Our primary goal was to understand what components could be added to an informal STEM family engagement program to best improve parent involvement. The museum educators in this study previously developed the family education workshops with bilingual (Spanish/English) families experiencing poverty (Garibay, 2007). The position of the museum facilitators was as a community partner that sought to broaden access to informal STEM learning for children experiencing poverty. The museum worked with researchers to evaluate two research questions (RQ) about the basic, core family engagement program and two additive conditions theorized to increase parent involvement.

RQ1: To what extent did families attend the core treatment activity of *TT STEM* workshops and did participation vary by background characteristics?

RQ2: Which conditions better increased parent involvement in STEM activities with their child?

We hoped for > 75% attendance at workshops, but past *Teaching Together* studies with families experiencing poverty showed an average of 25% attendance (Zucker et al., 2021). We hypothesized that parents in all treatment conditions would report increased involvement in STEM, but that parents who received the take-home kits would report more frequent STEM because providing materials reduced barriers. We expected adding contingent monetary rewards would further boost parent involvement because it reinforced the value of doing STEM.

## Method and materials

This study occurred in the 2019–2020 school year in a south-central U.S. state within 20 schools where 92% of students received free/reduced lunch. We used a cluster randomized control trial design, randomly assigning conditions at the school level to: BAU control, Treatment A/Core, Treatment B/Add Materials, and Treatment C/Add Incentives. All pre-k families in participating schools were invited and written consent was required (IRB # HMC-MS-15-0759). The study was advertised using flyers in school-home communication folders or parent meetings hosted at the school. Amongst consented families, we randomly selected an average of four parent–child dyads per classroom (SD = 2.32), totaling 181 parent–child dyads. Due to 17 families completing the pretest survey after treatment started and attrition at posttest, 123 families represent the final sample. Table 1 shows demographics. Mean child age was 4 years and 5 months (SD = 0.34 months; range 3 years, 5 months to 5 years, 0 months); 51% were female. Most participants were Black or Hispanic/Latine. About 50% of families spoke a language other than English at home (*n* = 88, 63% Spanish). Median yearly household income was $20,001–$30,000. Families received $50 for completing assessments in Fall/baseline, $50 for Summer/posttest, and $20 in Winter/follow-up. Testing occurred September–November 2019 for pre-k baseline, May–July 2020 for pre-k posttest and January–March 2021 for kindergarten (K) follow-up. Detailed participant demographics, attrition analysis, and CONSORT flowchart are in Online Supplementary materials SM1, SM2. Treatment activities were explained above, but sample materials and cost analysis are in Online Supplementary materials SM3–SM7. Parents reported high satisfaction with workshops (*M* = 3.84, SD = 0.47) on a 4-point scale at workshop exit surveys. Families in the control group experienced their school’s BAU family engagement offerings and a set of developmental text messages from the researchers to maintain contact/reduce attrition; this is detailed in Online Supplementary material SSM8.

**Table 1:** Demographic characteristics (*n* =  123).

	C (*n* = 37)	TxA (*n* = 15)	TxB (*n* = 37)	TxC (*n* = 34)
Child female?	0.59 (0.50)	0.33 (0.49)	0.54 (0.51)	0.56 (0.50)
Other language at home?	0.30 (0.46)	0.53 (0.52)	0.68 (0.47)	0.71 (0.46)
Mother’s level of education	4.51 (1.73)	4.53 (1.55)	4.54 (2.05)	4.82 (1.47)
Father’s level of education	3.46 (1.24)	4.93 (1.94)	3.69 (2.00)	4.34 (2.13)
Is caregiver Hispanic?	0.25 (0.44)	0.40 (0.51)	0.47 (0.51)	0.45 (0.51)
Caregiver race				
Black	0.70 (0.46)	0.47 (0.52)	0.49 (0.51)	0.29 (0.46)
White	0.08 (0.28)	0.33 (0.49)	0.32 (0.47)	0.38 (0.49)
Household income	3.35 (1.81)	4.36 (1.21)	3.63 (1.59)	3.59 (1.91)

C, Control; TxA, Core program; TxB, Add kits; TxC = Add rewards.

### Measures

The primary outcome was a 10-item parent involvement survey collected at baseline, pre-k posttest, and kindergarten follow-up. Responses ranged from: *1*-Not at all; *2*-Once or twice a week; *3*-Three or more times a week, but not everyday; to *4*-Everyday. Items asked “How many times in the past week have you…” around STEM activities such as “compared sizes of objects or toys with your child?” “talked to your child about plants, animals or other living things?” These items were adapted from the Head Start Family and Child Experiences Survey (West et al., 2007). Sample reliability was Cronbach’s α = 0.85. Online Supplementary Table SM9 shows descriptives and all items in the parent involvement survey.

### Data analysis plan

To answer RQ1, we used descriptive statistics to group families into groups of non-attenders, lower, and higher attenders. We then explored the statistical significance of these levels using the Kruskal-Wallis test, a non-parametric one-way ANOVA.

To examine RQ2, we first estimated the intent-to-treat (ITT) using ordinary least squares regressions, correcting for clustering using robust standard errors at the classroom and school-level. Model 1 regressed the outcome on the baseline and three treatments (control as reference). Model 2 added family-level demographic characteristics: child’s sex (male = 0; female = 1); language other than English at home (0 = no; 1 = yes); highest level parent education; number of parents in a STEM-related career (0 = none; 1 = one parent, 2 = two parents); race/ethnicity of parent with three dummy variables for White, Black, and Hispanic. For Model 3, we added school-level variables from the Texas Education Agency 2019–2020 school profile reports: percent economically disadvantaged students, percent Limited English Proficiency students, and percent special education students. We also report treatment-on-treated (TOT) estimates by dividing the ITT estimates by the percent of treatment group members who were treated, defined as attending at least one workshop. This adjustment is appropriate given there were no cross-overs in our experiment (only no-shows). We had minimum levels of missing data on family-level covariates in Models 1 and 2. We used a multiple imputation approach to missing data.

## Results

### RQ1-attendance patterns

Across all groups, we had rather low, average 25% attendance rates (*M* = 1.5 workshops, SD = 1.7, range = 16–36%). Rates were 40% for Treatment A, 65% for Treatment B, and 56% for Treatment C. Parents reported the most salient barriers to attendance were limited time due to competing work/family priorities (Supplementary Table SM10). The pattern of attendance, shown in Online Supplementary Figure SM2, shows parent attendance improved at workshops 2 through 4 but, at workshops 5 and 6, attendance was lower. Descriptively, we looked at characteristics of families most likely to attend the workshops. To this end, we categorized attendance into five groups: *Group 0* had families who attended no STEM workshops (*n* = 60); *Group 1* families attended <= 25% (*n* = 22); *Group 2* families attended between > 25% and < = 50% (*n* = 24); *Group 3* families attended > 50% and < = 75% (*n* = 16); *Group 4* families attended > 75% of offered workshops (*n* = 15). Table 2 reports background characteristics by descriptive group. Families that attended > 50% of workshops had higher levels of mother’s education and father’s education, higher proportion of White parents, and higher incomes than those families who attended less than half. The only significant characteristic at *p* < 0.05 was father’s education (*p* = 0.034). In the lower panel of Table 2, we connect these varying attendance rates to fixed costs of delivering workshops. This shows how the cost per school increases when fewer families attend due to largely fixed costs.

**Table 2:** Workshop attendance.

Background characteristics	Group 0: 0%	Group 1: ≤25%	Group 2: ≤50%	Group 3: ≤75%	Group 4: >75%	Kruskal–Wallis test
	(*n* = 60)	(*n* = 22)	(*n* = 24)	(*n* = 16)	(*n* = 15)	
Mother’s highest education	4.43 (1.63)	4.67 (1.62)	3.88 (1.62)	4.86 (1.92)	4.73 (2.05)	*χ*^2^(*df* = 4) = 5.14, *p* = 0.273
Father’s highest education*	4.15 (1.76)	4.20 (1.77)	3.17 (1.61)	4.71 (2.70)	5.29 (2.40)	*χ*^2^ (*df* = 4) = 10.39, *p* = 0.034*
Mother STEM related	0.33 (0.48)	0.35 (0.49)	0.26 (0.45)	0.50 (0.52)	0.13 (0.35)	*χ*^2^ (*df* = 4) = 4.96, *p* = 0.291
Father STEM related	0.39 (0.49)	0.29 (0.47)	0.45 (0.51)	0.64 (0.50)	0.36 (0.50)	*χ*^2^(*df* = 4) = 4.42, *p* = 0.352
Home language other than English	0.48 (0.50)	0.43 (0.51)	0.71 (0.46)	0.60 (0.51)	0.73 (0.46)	*χ*^2^ (*df* = 4) = 6.88, *p* = 0.143
Hispanic caregiver	0.33 (0.48)	0.30 (0.47)	0.48 (0.51)	0.53 (0.52)	0.40 (0.51)	*χ*^2^ (*df* = 4) = 3.48, *p* = 0.481
Race caregiver						
Black	0.55 (0.50)	0.57 (0.51)	0.29 (0.46)	0.40 (0.51)	0.33 (0.49)	*χ*^2^ (*df* = 4) = 6.85, *p* = 0.144
White^+^	0.21 (0.41)	0.24 (0.44)	0.38 (0.49)	0.47 (0.52)	0.53 (0.52)	*χ*^2^ (*df* = 4) = 9.04, *p* = 0.060
Household Income	3.46 (1.88)	3.28 (1.45)	3.24 (1.81)	4.08 (2.10)	3.92 (1.44)	*χ*^2^ (*df* = 4) = 2.58, *p* = 0.631
Treatments (Tx)						
TxA	0.32 (0.47)	0.27 (0.46)	0.29 (0.46)	0.13 (0.34)	0.13 (0.35)	*χ*^2^ (*df* = 4) = 3.85, *p* = 0.427
TxB	0.35 (0.48)	0.50 (0.51)	0.29 (0.46)	0.50 (0.52)	0.67 (0.49)	*χ*^2^ (*df* = 4) = 7.53, *p* = 0.110
TxC	0.33 (0.48)	0.23 (0.43)	0.42 (0.50)	0.38 (0.50)	0.20 (0.41)	*χ*^2^ (*df* = 4) = 3.13, *p* = 0.536
Cost Analysis (if *n* families attend)	*n* = 0	*n* = 6	*n* = 11	*n* = 17	*n* = 22	
Workshop fixed costs per school^a^	$1,879.87	$341.79	$170.90	$113.93	$85.45	

^+^*p* < 0.10; *p < 0.05; **p < 0.01; and ***p < 0.001.

a This does not include the variable cost of family museum passes valued at up to $84; this is the only variable treatment A/core costs, as all other costs are fixed.

### RQ2-conditions best increasing parent involvement

Table 3 presents three model specifications described above for ITT and TOT. There were no statistically significant associations, thus we interpret models based on effect sizes of TOT. The most robust Model 3, which adjusts for both family and school characteristics before comparing treatments to control, found at pre-k posttest that Treatment A and B produced no meaningful differences in parent involvement (TxA ES = −0.01; TxB ES = −0.08). But Treatment C higher pre-k posttest parent involvement compared to control (ES = 0.18).

**Table 3:** Parent involvement models comparing treatment (Tx) groups to control.

	Model 1					Model 2					Model 3				
	ITT	Robust standard error	Value of *p*	TOT	Effect size for TOT	ITT	Robust standard error	Value of *p*	TOT	Effect size for TOT	ITT	Robust standard error	Value of *p*	TOT	Effect size for TOT
Posttest, *n* = 123															
TxA	−0.15	0.18	0.419	−0.36	−0.57	−0.09	0.17	0.594	−0.23	−0.36	0.00	0.17	0.989	−0.01	−0.01
TxB	−0.25	0.15	0.095^+^	−0.39	−0.61	−0.19	0.15	0.229	−0.29	−0.46	−0.03	0.13	0.802	−0.05	−0.08
TxC	−0.16	0.11	0.161	−0.29	−0.46	−0.07	0.11	0.529	−0.13	−0.20	0.07	0.12	0.583	0.12	0.18
Follow-up, *n* = 74															
TxA	−0.29	0.15	0.063^+^	−0.63	−0.94	−0.09	0.17	0.586	−0.20	−0.30	−0.29	0.18	0.108	−0.63	−0.94
TxB	−0.15	0.21	0.484	−0.24	−0.36	−0.05	0.23	0.830	−0.08	−0.12	0.14	0.19	0.478	0.23	0.34
TxC	−0.07	0.16	0.678	−0.10	−0.15	0.00	0.20	0.986	0.01	0.01	0.00	0.18	0.987	0.00	−0.01

ITT, intent-to-treat; TOT, treatment-on-the-treated. ^+^*p* < 0.10; ^*^p < 0.05; ^**^p < 0.01; and ^***^p < 0.001.

The results for the delayed, follow-up K outcomes (lower panel Table 3) were, again, non-significant but the pattern of ES differed from pre-k posttest. For Model 3, Treatment A had substantially lower levels than control (ES = −0.94), Treatment B was higher than control (ES = 0.34), and Treatment C was similar to control (ES = −0.01). Parent surveys indicated the most salient barriers to parent involvement in STEM were limited time, limited materials/resources, and knowledge of how to support early STEM (Supplementary Table SM10).

## Discussion

This study explored informal learning conditions that are most likely to increase parent involvement in STEM with their young child. We randomly assigned schools to a control condition or one of three additive treatment groups with museum-led STEM workshops within school facilities as the core component. We added take-home activity kits and parent rewards in the other treatments. There were no significant impacts of any treatment on the primary outcome of parent involvement in STEM. Treatment A/Core program showed no difference at pre-k posttest but the largest and negative difference at K follow-up. Treatment B/Add Materials showed no difference at posttest but a moderate, non-significant positive difference at follow-up. Treatment C/Add Incentives showed a small positive difference at posttest, but no difference at follow-up. In other words, Treatment C’s monetary rewards for parents showed promise for short-term outcomes, but benefits faded over time. We consider potential explanations for the larger effect sizes of Treatments B and C that added materials to support STEM learning at home. These treatment findings and attendance patterns have implications for broader family engagement approaches.

Parent involvement is linked to children’s academic achievement (Castro et al., 2015; Ma et al., 2016). Although we found no significant effects of the *TT STEM* program, proving take-home family kits produced larger effect sizes. This is similar to prior reports that providing pre-k families experiencing poverty with access to typical family engagement programs may not be sufficient (Puma et al., 2010; Grindal et al., 2016). Like other studies that provide pre-k families with treatment packages that include home materials and others supports (Clements and Sarama, 2008; Welsh et al., 2020), this study found that families benefited most from conditions that included the take-home STEM kits. The contribution of this study is that we unbundled treatment packages to understand added benefits of different components. Interestingly, adding rewards in Treatment C improved immediate parent involvement (ES = 0.18), but these benefits faded by kindergarten follow-up when only Treatment B with take-home kits showed sustained improvement in parent involvement (ES = 0.34). Because all activity kits were delivered at the outset of the intervention, it is possible that providing kits allowed parents to build more culturally-relevant engagement strategies in their home than Treatment A that used a more traditional school-to-home approach of attending workshops to increase parent involvement (*cf.* McWayne et al., 2022).

Provision of STEM learning materials to families experiencing poverty warrants future consideration. We expect that providing materials alone, without education workshops and resources, will be ineffective (e.g., Neuman, 2017). Yet the lack of significant differences may be due to several factors. The limited scope of the program may not have developed broad and deep interest in informal STEM over multiple stages of development (National Research Council, 2009). Indeed, some effective STEM approaches using take-home materials span several grade levels (Kaderavek et al., 2020) or ensure many museum visits (Pattison et al., 2020). Yet other, intensive parent coaching studies that intervene in pre-k and kindergarten find sustained effects on parent involvement through Grade 5 (ES = 0.24; Welsh et al., 2020). Future studies should tease apart issues of intensity of parent involvement supports needed across grades as well as the extent to which step-by-step kits versus more open-ended materials for STEM exploration are beneficial over time.

The finding that the benefits of the added monetary rewards condition faded when they were withdrawn at the kindergarten follow-up survey, aligns with theories that performance-based extrinsic rewards have proximal influences on behaviors adults already hoped and intended to do (Parker et al., 2017). For example, the rewards may have urged parents to overcome immediate time pressures supporting STEM learning; parents noted limited time was their primary barrier to involvement in STEM. This aligns with a recent pre-k shared book reading study that found the most effective short-term technique for encouraging parents to read with their child was paying parents $0.50 for each book reading session (Justice et al., 2018). Justice and colleagues concluded that rewards can support parent involvement particularly when time pressures are a salient barrier.

Although parents reported high satisfaction with the *TT STEM* workshops, they only attended an average of 25% of offered workshops. It is possible that these satisfaction data are overestimated because, out of respect for perceived museum experts, parents reported that the events were engaging and useful; this is common when families perceive a hierarchical relationship (McWayne et al., 2022). Other family engagement studies show families complete 35–75% of offered activities (Justice et al., 2018; Kim et al., 2019; Welsh et al., 2020). We found significantly higher workshop attendance for families with higher paternal education levels and trends for higher income and White families attending more events. These findings are troubling in that the families experiencing poverty and racial/ethnic minorities were the target populations for our goal of broadening access to early STEM opportunities (National Research Council, 2009). This could suggest the *TT STEM* program was not sufficiently tailored to the needs of these populations. Alternatively, there may be an upper limit to the number of workshops in-person parents can attend. In future studies, we will consider flexible or adaptive options to improve uptake (*cf.* Kim et al., 2019). Our cost analysis findings are noteworthy because they show how the fixed costs of family education workshops move from costs per student from $85 if all families in a classroom attend to $342 if only 25% attend. This has implications for other family engagement programs to consider how to schedule and market events to ensure high attendance (Beckett et al., 2009).

### Limitations

There are shortcomings of this study to note. First, we did not measure child outcomes. Second, this sample likely was underpowered to detect potentially meaningful effects. Third, a small number of workshops were cancelled due to local emergencies or the start of the COVID-19 pandemic. The pandemic could have impacted the reliability of our parent involvement survey. Finally, there was greater attrition than desired including low response rates on the kindergarten parent surveys. These limitations limit the conclusions we can draw from these data.

### Conclusion

These patterns of findings for parent involvement align with meta-analyses that light touch educational workshops produce null to small impacts (Grindal et al., 2016). Yet the results demonstrate that families experiencing poverty can be better supported to engage in early STEM activities with their young children under certain conditions. That is, consistent with past research (Boonk et al., 2018), giving families access to educational resources alongside materials that scaffold informal learning were the most beneficial treatments for improving parent involvement in this sample. This is important for other programs with goals of promoting broad access to informal learning in ways that ensure access to families experiencing poverty.

## Data availability statement

The raw data supporting the conclusions of this article will be made available by the authors, without undue reservation.

## Ethics statement

The studies involving human participants were reviewed and approved by Committee for the Protection of Human Subjects (CPHS) at University of Texas Health Science at Houston, Medical School (MS). Written informed consent to participate in this study was provided by the participants' legal guardian/next of kin. Written informed consent was obtained from the individual(s) for the publication of any identifiable images or data included in this article.

## Author contributions

TZ: conceptualization, qualitative analysis, resources, writing original draft, writing—reviewing and editing, visualization, supervision, and funding acquisition. GM: conceptualization, quantitative analysis, writing original draft, writing—reviewing and editing, visualization, and data curation. MA: conceptualization, supervision, and writing—reviewing and editing. CM: supervision, writing reviewing and editing, funding acquisition, and project administration. CE: supervision, writing—reviewing and editing, and project administration. JS and LL: data curation, visualization, and cost analysis. All authors contributed to the article and approved the submitted version.

## Funding

Research reported in this publication was supported by Advancing Informal STEM Learning division of the National Science Foundation under award number 1811356.

## Conflict of interest

The authors declare that the research was conducted in the absence of any commercial or financial relationships that could be construed as a potential conflict of interest.

## Publisher’s note

All claims expressed in this article are solely those of the authors and do not necessarily represent those of their affiliated organizations, or those of the publisher, the editors and the reviewers. Any product that may be evaluated in this article, or claim that may be made by its manufacturer, is not guaranteed or endorsed by the publisher.
