# Supplementary material for: Informal science, technology, engineering and math learning conditions to increase parent involvement with young children experiencing poverty
Source: Front Psychol. 2022 Nov 1;13:1015590. doi: 10.3389/fpsyg.2022.1015590 (PMC9683104; doi:10.3389/fpsyg.2022.1015590)
Supplement: Supplementary file 1 [file Data_Sheet_1.docx]

# Online Supplemental Appendix

## These materials accompany the article entitled, *Informal STEM Learning Conditions to Increase Parent Involvement with Young Children Experiencing Poverty*.

| **Table SM1** |  |  |  |  |  |  |  |  |  |  |
| --- | --- | --- | --- | --- | --- | --- | --- | --- | --- | --- |
| *Participant Baseline Demographic Characteristics and Balance Check for Posttest Analytic Sample (n = 123) and Kindergarten Follow-up Analytic Sample (n = 74); Means and (Standard Deviations)* | | | | | | | | | | |
|  |  |  | Posttest Analytic Sample (*n* = 123) | | |  | Kindergarten Follow-up Sample (*n* = 74) | | | |
|  | BAU  (*n* = 37) | TxA  (*n* = 15) | TxB  (*n* = 37) | TxC  (*n* = 34) | *F*-test for continuous variables;  Chi-Square for count/categorical | BAU  (*n* = 21) | TxA  (*n* = 13) | TxB  (*n* = 18) | TxC  (*n* = 22) | *F*-test for continuous variables;  Chi-Square for count/categorical |
| Demographic and Family Characteristics | | | | | | | | | | |
| Child Female? | 0.59  (0.50) | 0.33  (0.49) | 0.54  (0.51) | 0.56  (0.50) | χ2(3) = 3.06; *p* = .382 | 0.67  (0.48) | 0.38  (0.51) | 0.72  (0.46) | 0.59  (0.50) | χ2(3) = 4.04; *p* = .257 |
| Other language at home? | 0.30  (0.46) | 0.53  (0.52) | 0.68  (0.47) | 0.71  (0.46) | χ2(3) = 15.23; *p* = .002 | 0.33  (0.48) | 0.62  (0.51) | 0.67  (0.49) | 0.64  (0.49) | χ2(3) = 5.87; *p* = .118 |
| Mother's highest level of  education | 4.51  (1.73) | 4.53  (1.55) | 4.54  (2.05) | 4.82  (1.47) | *F*(3, 38) = 0.24; *p* = .870 | 4.62  (1.63) | 4.54  (1.66) | 4.56  (1.92) | 4.43  (1.33) | *F*(3, 34) = 0.08; *p* = .973 |
| Father's highest level of  education | 3.46  (1.24) | 4.93  (1.94) | 3.69  (2.00) | 4.34  (2.13) | *F*(3, 38) = 2.54; *p* = .071 | 3.62  (0.33) | 5.00  (2.00) | 3.59  (2.09) | 3.85  (2.03) | *F*(3, 34) = 1.46; *p* = .244 |
| Mother STEM-related career | 0.36  (0.49) | 0.36  (0.50) | 0.44  (0.50) | 0.28  (0.46) | χ2(3) = 1.83; *p* = .609 | 0.33  (0.48) | 0.33  (0.49) | 0.40  (0.51) | 0.25  (0.44) | χ2(3) = .91; *p* = .823 |
| Father STEM-related career | 0.51  (0.51) | 0.62  (0.51) | 0.31  (0.47) | 0.43  (0.50) | χ2(3) = 4.50; *p* = .212 | 0.63  (0.50) | 0.58  (0.51) | 0.20  (0.41) | 0.37  (0.50) | χ2(3) = 7.70; *p* =.053 |
| Mother+Father STEM-related  career | 0.84  (0.80) | 0.93  (0.83) | 0.71  (0.79) | 0.67  (0.82) | *F*(3, 38) = 0.63; *p* = .601 | 0.90  (0.77) | 0.92  (0.90) | 0.56  (0.73) | 0.57  (0.74) | *F*(3, 34) = 0.97; *p* = .420 |
| Is caregiver Hispanic? | 0.25  (0.44) | 0.40  (0.51) | 0.47  (0.51) | 0.45  (0.51) | χ2(3) = 4.57; *p* = .207 | 0.33  (0.48) | 0.46  (0.52) | 0.59  (0.51) | 0.48  (0.51) | χ2(3) = 2.50; *p* = .474 |
| Caregiver race |  |  |  |  |  |  |  |  |  |  |
| Black | 0.70  (0.46) | 0.47  (0.52) | 0.49  (0.51) | 0.29  (0.46) | χ2(3) = 11.932; *p* = .008 | 0.67  (0.48) | 0.38  (0.51) | 0.39  (0.51) | 0.32  (0.48) | χ2(3) = 6.03; *p* = .110 |
| White | 0.08  (0.28) | 0.33  (0.49) | 0.32  (0.47) | 0.38  (0.49) | χ2(3) = 9.773; *p* = .021 | 0.10  (0.30) | 0.38  (0.51) | 0.44  (0.51) | 0.41  (0.50) | χ2(3) = 7.15; *p* = .067 |
| Household income | 3.35  (1.81) | 4.36  (1.21) | 3.63  (1.59) | 3.59  (1.91) | *F*(3, 36) = 1.53; *p* = .223 | 3.50  (1.79) | 4.11  (1.17) | 3.59  (1.59) | 3.24  (2.05) | *F*(3, 30) = 0.89; *p* = .457 |
| Baseline measures | | | | | | | | | | |
| Parent involvementa | 2.79  (0.63) | 2.65  (0.68) | 2.72  (0.49) | 2.64  (0.54) | *F*(3, 38) = 0.59; *p* = .624 | 2.89  (0.69) | 2.69  (0.69) | 2.78  (0.47) | 2.53  (0.50) | *F*(3, 34) = 2.04; *p* = .127 |
| *Notes*. For the posttest sample (end of intervention), the analytic sample is defined as those parents who completed a posttest survey at the end of intervention and who completed a pretest before they workshop. For the follow-up sample (beginning of kindergarten), the analytic sample is defined as those parents who completed a follow-up survey and who completed a pretest before they attended a workshop.aRanges from 1=none to 4 everyday; BAU = Control; TxA =Treatment A (Core Program); TxB =Treatment B (Add take home STEM kits); TxC: Treatment C (Add rewards) | | | | | | | | | | |

# Figure SM1

*Consort Flow Diagram*


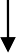


Excluded (n=168)

- Not meeting inclusion criteria (n=12)
- Declined to participate (n=81)
- Difficult to schedule (n=51)
- No response to invitation (n= 24)

**Treatment C (n=44)**

Parent Surveys (n=34; n=29 for true pretest)

**Control (n=44)**

**Treatment B (n=57)**

**Treatment A (n=36)**

*Parent Surveys (n=176; 160 with true pretest)

**Pre-Assessment**

Randomized (n=181)

Total # Invited (n= 1,012)

**Enrollment**

**Post-Assessments**

Analytic sample: Completed partial post- test parent survey and has true pretest assessment (n=15)

Analytic sample: Completed partial post- test parent survey and has true pretest assessment (n=37)

Parent Surveys (n=43; n=43 for true pretest)

Parent Surveys (n=44; n=40 for true pretest

Parent Surveys (n=55; n= 48 for true pretest)

**
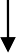
**

Analytic sample: Completed partial post- test parent survey and has true pretest assessment (n=37)

Analytic sample: Completed partial post- test parent survey and has true pretest assessment (n=34)

| **Table SM2** |  |  |  |  |  |
| --- | --- | --- | --- | --- | --- |
|  |  |  |  |  |  |
| *Differences between Attriters (n = 41) and non-Attriters (n = 140); Means and (Standard Deviations) at Posttest* | | | | | |
|  | Attriters  (n = 41) | Non-  Attriters  (n = 140) | Unstandardized regression coefficient  (Attriters - Non-attriters) | Difference  as Effect Size | *p*-value |
| Demographic and Family Characteristics | | | | | |
|  | 0.46 | 0.53 |  |  |  |
| Child Female? | (0.50) | (0.50) | -0.03 | -0.06 | 0.578 |
|  | 0.36 | 0.54 |  |  |  |
| Other language at home? | (0.49) | (0.50) | -0.13 | -0.27 | 0.074 |
|  | 3.67 | 4.65 |  |  |  |
| Father's highest level of education | (1.28) | (1.75) | -0.06 | -0.03 | 0.000 |
|  | 3.62 | 4.04 |  |  |  |
| Mother's highest level of education | (1.57) | (1.93) | -0.02 | -0.02 | 0.130 |
|  | 0.26 | 0.36 |  |  |  |
| Mother STEM-related career | (0.45) | (0.48) | -0.08 | -0.16 | 0.329 |
|  | 0.38 | 0.45 |  |  |  |
| Father STEM-related career | (0.49) | (0.50) | -0.04 | -0.09 | 0.526 |
|  | 0.61 | 0.77 |  |  |  |
| Mother+Father STEM-related career | (0.72) | (0.80) | -0.05 | -0.06 | 0.323 |
|  | 0.31 | 0.38 |  |  |  |
| Is caregiver Hispanic? | (0.46) | (0.49) | -0.05 | -0.16 | 0.441 |
| Caregiver race |  |  |  |  |  |
|  | 0.54 | 0.50 |  |  |  |
| Black | (0.51) | (0.50) | 0.02 | 0.05 | 0.752 |
|  | 0.18 | 0.28 |  |  |  |
| White | (0.39) | (0.45) | -0.09 | -0.34 | 0.163 |
|  | 2.81 | 3.62 |  |  |  |
| Household income | (1.80) | (1.73) | -0.05 | -0.03 | 0.010 |
| Baseline measures | | | | | |
| Parent involvementa | 2.63 | 2.69 |  |  |  |
|  | (1.80) | (0.56) | -0.03 | -0.05 | 0.575 |

Note. Attriters are defined as those parents who did not complete a post-survey. Unstandardized regression coefficients were obtain from a regression predicting attriter status, adjusting standard errors for clustering at the classroom-level.

aRanges from 1=none to 4 everyday

Education was measured as an 8-category variable ranging from 1 to 8 where 1 represents <=8th grade and 9 = professional degree. Household income was measured as an 8-category variable ranging from 1 (11K or less) to 8 ($150K or more). For the combined mother and father STEM-related career variable, 0 = none of the parents have a STEM-related career, 1 = one of the parents has a STEM-related career, and 2 = both parents have a STEM-related career.

Overall F-test where all variables listed in table were used to predict attrition was statistically significant, F(15,41) = 3.64, p = .0005.

# Table SM3

*Sample Core Treatment Workshop Event Photos*

| Event (Activity Station) | Image |
| --- | --- |
| Workshop 1: What’s the Big Idea? (Water Drop Art Activity) | 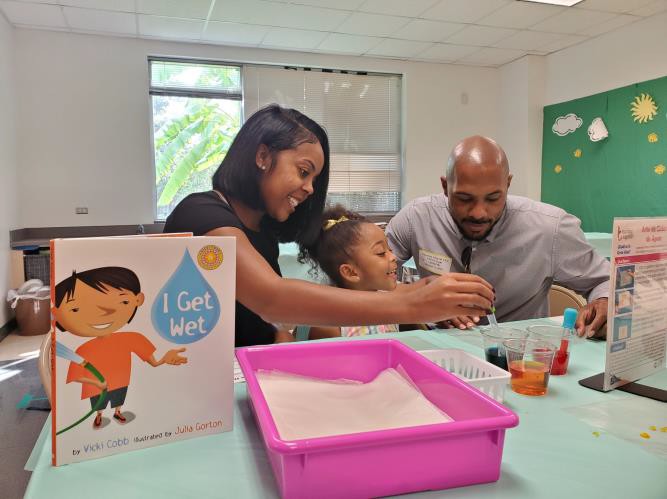 |
| Workshop 2: Let’s Figure It Out! (Boats Afloat Activity) | 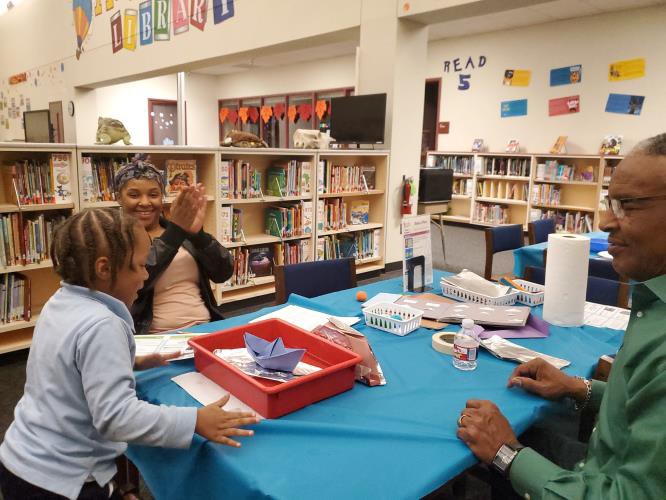 |
| Workshop 2: Let’s Figure It Out (Sticky Situation Activity) | 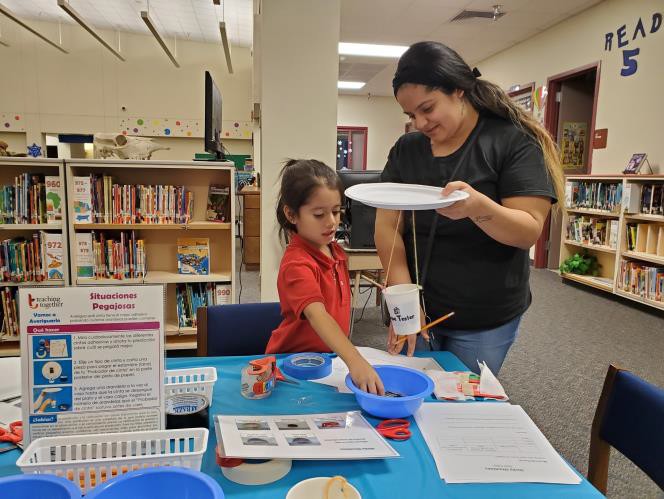 |

# Table SM4

Overview of the Teaching Together STEM Components by Treatment Conditions

| **Conditions** | **Description** |
| --- | --- |
| Treatment A – Core TT STEM | Six in-person workshops:  **1. “What’s the Big Idea”** STEM Language: Parents were introduced to the overarching concept that you can increase your child’s curiosity about the world through rich conversations that include asking open-ended questions and routinely explaining technical and scientific vocabulary.  **2. “Let’s Figure It Out”** STEM Inquiry: Parents learned how to explore science concepts in everyday activities using reasoning, inquiry, predictions, and experimentation strategies.  **3. “Math Rules!”** Early Math: Parents learned how to integrate counting, number identification, and comparison talk into everyday activities.  **4. “Show What You Know”** Gather and Analyze Data: Parents explored how to gather information as you interact with their child to include counting, tallies, and charts as simple forms of data in daily life.  **5. “Dream It, Build It!”** Engineering: Parents were presented with ways to encouraging tinkering and creative problem solving within playful activities and common household materials.  **6. “Picture This”** Systems and Models: Parents and children explored patterns and systems in the world such as , seasons, plants, and the human body. |
|  | Text messages:  **Event reminders** sent 1 week before and 2 days before event with location and time  **Strategy explanations** sent 1 day and 8 days after event. These are 24 informational tips for parents and conversation starters linked to workshop theme.  **Activity video** links sent 4 days and 12 days after event. Short text message invited parents to click for a fun, informal learning activity using common household objects (not kits) |
| Treatment B – Add Kits | English or Bilingual English/Spanish kits:  **Kit 1**: Ocean animals floor puzzle and book *Hello Ocean* by Pam Muñoz Ryan  **Kit 2**: Sink or float activity with foam pieces and weights to build a boat and test  **Kit 3**: Counting butterflies board game  **Kit 4**: Plastic insect counting manipulatives and *How Many Snails?: A Counting Book* by Paul Giganti  **Kit 5**: Plastic links for non-standard measurement of household objects  **Kit 6**: Wheels, axels, foam pieces and other materials to build balloon-powered cars  **Kit 7**: Tangram pieces and sample puzzles to create shapes  **Kit 8**: Plastic blocks and vehicle building pieces with photos of sample vehicles  **Kit 9**: Animal life cycle manipulatives for frog, chicken, or butterfly. One matching title was given *From Tadpole to Frog* by Wendy Preffer or *Where do Chicks Come from*? by Amy E. Sklansky or *From Caterpillar to Butterfly* by Deboarah Heiligman |
| Treatment C – Add Rewards | Opportunity for six reward payments:  **$2.50 reward** payment if parent sent researchers a text message or photo related to any learning activity they did recently with their child |

**Table SM5**

*Sample Parent Text Messages: Event Reminders & Tips*

| **Text Type** | **Text Content** |
| --- | --- |
| Intro | Part 1: Hi [first_name]! Welcome to Teaching Together STEM! You received this message because you signed up to participate in a research study with [University] and the Children's Museum [Location]. 📝 (U+1F4DD)  Part 2: You’ll receive text messages ✉️ (U+2709) during the year to invite you to workshops with pizza 🍕 (U+1F355) at your child's school. Other messages will explain science, technology, engineering and math (STEM) activities to help [child_first_name] get ready for kindergarten (K). 🏫 (U+1F3EB)  Part 3: If you don’t want these messages? Reply STOP to Quit/HELP for Info/Msg&DataRatesMayApply. |
| Event1a | Event Reminder: Come on [event1_date] at [event1_time] to a family event at [child_first_name]'s school 🎉 (U+1F389). Join the Children's Museum for a workshop called "What's the Big Idea?" to discover ways to unlock your child's curiosity about the world. Includes pizza 🍕 (U+1F355) and a family museum pass |
| Event1b | Event Reminder: Don’t forget to join us on [event1_date] at [event1_time] to a family event at [child_first_name]'s school 🎉 (U+1F389). Includes pizza (U+1F355) and a family museum pass. |
| Activity 1 | Encourage your child to ask questions. Follow this link for a fun activity: https://cliengagefamily.org/bug-photo-hunt/ |
| Tip2 | Teach Big Words 📖 (U+1F4D6) - Help [child_first_name] get ready for Kinder by teaching [him_her] big words with simple explanations. For example, say, “When we cook, we use cooking equipment.” |
| Activity 2 | Using big words can help build your child's vocabulary. Follow this link for a fun activity: https://cliengagefamily.org/sensory-popcorn/ |
| Question1^a^ | Tell us about an activity you did with [child_first_name] this week to support [his_her] learning. Include a photo 📷 (U+1F4F7) if you like. Response to any: Thank you for your response.  Non-responsive: +48 hrs  Just a reminder … Tell us about an activity you did with [child_first_name] this week to support [his_her] learning. |

*Note*. Child name/gender/date personalization and emoji commands shown in brackets

^a^Response rates by Group: 7 parents (19.44%) in Treatment A; 23 (40.353%) in Treatment B; 21 parents (47.73%) parents in Treatment C; and 12 parents (27.27%) in the control group.

# Table SM6

*Count of Workshops Offered by Treatment Group*

| **Workshops** | | |
| --- | --- | --- |
| **Treatment A Schools**  *M* = 4.8 workshops (*SD* = 1.19, min = 3, max = 6) | Offered | Missing a |
| School 1 | 6 | 0 |
| School 2 | 6 | 0 |
| School 3 | 4 | 2 |
| School 4 | 4 | 2 |
| School 5 | 3 | 3 |
| **Treatment B Schools**  *M =* 5.2 workshops (*SD* = 0.76, min = 4, max = 6) | | |
| School 6 | 6 | 0 |
| School 7 | 6 | 0 |
| School 8 | 5 | 1 |
| School 9 | 5 | 1 |
| School 10 | 4 | 2 |
| **Treatment C Schools**  *M =* 4.2 workshops (*SD* = 1.63, min = 2, max = 6) | | |
| School 11 | 6 | 0 |
| School 12 | 6 | 0 |
| School 13 | 4 | 2 |
| School 14 | 3 | 3 |
| School 15 | 2 | 4 |
| **Totals** | **70** | **20** |
| a19 of the missing workshops were cancelled due to local/facilities problems within a school or to COVID-related school closures.  *Note*. Although teachers/school staff attendance was optional, there was a teacher/staff present in about 32% of all workshops (40% for treatment A; 33% for treatment B; 23% for treatment C). There was not a consistent relationship between teacher/staff attendance of the workshops and proportion of families that attended (*r* = .22, *p* = .438). | | |

**Table SM7**

*Cost Analysis for Teaching Together STEM Treatments*

|  |  | **Costs for 1 Workshop** | | **Costs for 6 Workshops** | |  |
| --- | --- | --- | --- | --- | --- | --- |
| **Ingredient** | **Description** | **Cost per school^1^** | **Cost per student^2^** | **Cost per school^1^** | **Cost per student^2^** | **Payer** |
| **Initial Workshop Startup Costs** |  |  |  |  |  |  |
| **Personnel** |  |  |  |  |  | Museum |
| Training: Content Expert (trainer) | Salary: $75,000 per year; 15 hr | $540.87 | $24.58 | $540.87 | $24.58 | Museum |
| Training: Facilitator (trainee) | Salary: $50,000 per year; 15 hr | $360.58 | $16.39 | $360.58 | $16.39 | Museum |
| Facilitator Time | Salary: $50,000 per year; 0.5 hr | $12.02 | $0.55 | $72.12 | $3.28 | Museum |
| *Personnel Costs Subtotal* | | $913.46 | $41.52 | $973.56 | $44.25 |  |
| **Materials & Equipment** |  |  |  |  |  |  |
| Workshop Activity Kits | 5 kits per workshop; $90 per kit | $450.00 | $20.45 | $2,700.00 | $122.73 | Museum |
| *Materials & Equipment Subtotal* | | $450.00 | $20.45 | $2,700.00 | $122.73 |  |
| **Recurring Workshop Costs** |  |  |  |  |  |  |
| **Personnel** |  |  |  |  |  |  |
| Organizing Materials for Family Fun Night | Salary: $50,000 per year; 0.5 hr | $12.02 | $0.55 | $72.12 | $3.28 | Museum |
| Review and Practice of Lesson | Salary: $50,000 per year; 2 hrs | $48.08 | $2.19 | $288.46 | $13.11 | Museum |
| Transferring Materials to Vehicle | Salary: $50,000 per year; 0.25 hr | $6.01 | $0.27 | $36.06 | $1.64 | Museum |
| Travel Time (to and from event) | Salary: $50,000 per year; 1 hr | $24.04 | $1.09 | $144.23 | $6.56 | Museum |
| Sign In/Meet School Contact | Salary: $50,000 per year; 0.25 hr | $6.01 | $0.27 | $36.06 | $1.64 | Museum |
| Room Arrangement/Straightening | Salary: $50,000 per year; 0.25 hr | $6.01 | $0.27 | $36.06 | $1.64 | Museum |
| Organization of Materials into stations | Salary: $50,000 per year; 0.75 hr | $18.03 | $0.82 | $108.17 | $4.92 | Museum |
| Amount of Time Allotted for Early Arrivals to Workshop | Salary: $50,000 per year; 0.25 hr | $6.01 | $0.27 | $36.06 | $1.64 | Museum |
| Presentation of Workshop Lesson | Salary: $50,000 per year; 1.25 hrs | $30.05 | $1.37 | $180.29 | $8.19 | Museum |
| Room Clean Up | Salary: $50,000 per year; 0.5 hr | $12.02 | $0.55 | $72.12 | $3.28 | Museum |
| Packing Materials Away/Transferring to Vehicle | Salary: $50,000 per year; 0.25 hr | $6.01 | $0.27 | $36.06 | $1.64 | Museum |
| Sanitizing and Organizing Materials | Salary: $50,000 per year; 2 hrs | $48.08 | $2.19 | $288.46 | $13.11 | Museum |
| Ordering Replacement Materials/Consumables | Salary: $50,000 per year; 1 hr | $24.04 | $1.09 | $144.23 | $6.56 | Museum |
| Track Attendance and Feedback | Salary: $50,000 per year; 0.75 hr | $18.03 | $0.82 | $108.17 | $4.92 | Museum |
| *Personnel Costs Subtotal* | | $264.42 | $12.02 | $1,586.54 | $72.12 |  |
| **Materials & Equipment** |  |  |  |  |  |  |
| Advertising | Flyers & Reminder Stickers; $0.86 | $18.92 | $0.86 | $113.52 | $5.16 | Researcher |
| *Materials & Equipment Subtotal* | | $18.92 | $0.86 | $113.52 | $5.16 |  |
| **Miscellaneous** |  |  |  |  |  |  |
| Text Messaging Service (event reminders, tips) | $5416.5 per year, up to 200 users | $27.08 | $1.23 | $27.08 | $1.23 | Researcher |
| Food: 8 slices per pizza, 2 slices per person | $12 per pizza; 3 people per student | $198.00 | $9.00 | $1,188.00 | $54.00 | Researcher |
| Facilitator Travel to Schools, mileage  reimbursement | $0.57 per mile; 14 miles round trip | $7.98 | $0.08 | $47.88 | $0.46 | Museum |
| Family museum admission pass^a^ | $84 (14 per person, for up to 6 guests) | $1,848.00 | $84.00 | $11,088 | $504.00 | Museum |
| *Miscellaneous Subtotal* | | $2,081.06 | $93.21 | $12,350.96 | $558.60 |  |
| **Treatment A Total** |  | $3,727.86 | $168.07 | $17,724.58 | $802.85 | Museum |
| **Treatment B Additions** |  |  |  |  |  |  |
| Family Take Home Kits^a^ | $155 per kit; 9 activities per kit | **$3,410.00** | **$155.00** | **$3,410.00** | **$155.00** | Researcher |
| **Treatment C Additions** |  |  |  |  |  |  |
| Family Rewards^a^ | $2.50 per text/photo, up to $40 | **$880.00** | **$40.00** | **$880.00** | **$40.00** | Researcher |
| ^1^Assumes 1 classroom per school  ^2^Assumes 22 students per classroom and 100% attendance  ^a^These variable costs increase as the number of families increases; all other costs are fixed costs for delivering the Core treatment components. | | | | | | |
|  |  |  |  |  |  |  |
|  |  |  |  |  |  |  |

**Table SM8**

*Business as Usual (BAU) Control Group Description*

| **Area** | **Activity Description** |
| --- | --- |
| BAU family engagement offerings | Parents rated as good at baseline (*M* = 5.93, *SD =* 1.66 scale *1* - not true at all to *7* - very true; sample item: “My child’s school provided activities for parents and children to do math/science at home.”). |
| Text Messages to Maintain Contact | Control group families received an equivalent number of 24 text messages as treatment families, but on non-STEM topics (e.g., safety, healthy hand hygiene, immunizations). Texts were personalized with the child’s first name, such as: “Sleep Habits - [child first name] needs 10-12 hours of sleep each night. Sleep can affect [child first name]’s growth and behavior.” |
| Family Museum Pass | At the end of the study/posttest visit, families received a family museum pass valued at up to $84 for admission of 6 guests to the local museum. |

| **Table SM9** |  |  |  |  |
| --- | --- | --- | --- | --- |
| *Descriptive Statistics for all Parent Involvement items in final analytic sample* (n=123) | | | | |
|  | Mean (SD) | | Percent of parents engaging daily with these activities | |
|  | Baseline | Posttest | Baseline | Posttest |
| **STEM-related items (primary outcome)** |  |  |  |  |
| In the past week, number of times you talked to your child about shapes | 3.04  (0.80) | 2.99  (0.88) | 31.97% | 34.15% |
| In the past week, number of times you compared sizes of different things with your child | 2.90  (0.79) | 2.85  (0.92) | 22.76% | 27.87% |
| In the past week, number of times you counted different things with child | 3.19  (0.78) | 3.11  (0.76) | 39.02% | 33.33% |
| In the past week, number of times you played counting games with your child | 3.10  (0.80) | 3.11  (0.86) | 36.07% | 38.52% |
| In the past week, number of times you talked with your child about how to make things move | 2.11  (0.91) | 2.24  (0.99) | 8.13% | 13.01% |
| In the past week, number of times you talked to your child about technology | 2.44  (0.92) | 2.71  (0.96) | 13.01% | 26.83% |
| In the past week, number of times you talked with your child about weather/seasons | 2.84  (0.99) | 2.87  (0.93) | 30.08% | 32.52% |
| In the past week, number of times that you talked with your child about nature-related topics | 2.57  (0.94) | 3.01  (0.83) | 18.85% | 33.33% |
| In the past week, number of times that you talked with your child about health-related topics | 3.06  (0.89) | 3.30  (0.83) | 37.40% | 50.41% |
| In the past week, number of times you played board/card games | 1.84  (0.87) | 2.02  (0.87) | 4.88% | 7.32% |
| **Literacy-related items** (not in outcome score, shown for comparison to STEM) |  |  |  |  |
| In the past week, number of times you talked to your child about writing (ranges from 1-4) | 3.06  (0.81) | 3.00  (0.80) | 32.52% | 30.08% |
| In the past week, number of times you read to your child (ranges from 1-4) | 3.15  (0.81) | 3.01  (0.83) | 38.21% | 32.52% |
| In the past week, number of times you taught your child letters (ranges form 1-4) | 3.06  (0.81) | Not asked | 49.59% | Not asked |

| **Table SM10** |  |  |
| --- | --- | --- |
| *Descriptive Statistics of Barriers to Parent Involvement and Workshop Attendance in Parent Surveys* | | |
|  | Baseline  (*n* = 123) | Posttest  (*n* = 123) |
|  | Mean (*SD*) | Mean (*SD*) |
| **Rating of Barriers to STEM involvement** (alpha = 0.84) |  |  |
| Not enough *time* to do math with child | 2.25 (1.62) | 2.43 (1.79) |
| Not enough *time* to do science with child | 2.15 (1.57) | 2.59 (1.83) |
| It takes *effort* for me to help my child succeed in math | 1.89 (1.43) | 1.98 (1.59) |
| It takes *effort* for me to help my child succeed in science | 2.08 (1.55) | 2.08 (1.59) |
| It takes effort for me to get the *materials* needed to do math with child | 2.30 (1.67) | 2.27 (1.75) |
| It takes effort for me to get the *materials* needed to do science with child | 2.39 (1.74) | 2.56 (1.78) |
| **Qualitative Themes Coded** (inter-rater agreement 97.8%, n=66 responses) Sample Responses | | |
| **Barriers to Workshop Attendance** | | |
| Limited time due to work/parent coursework (n=17) | “I work and attend school so I was tired some days.” | |
| Limited time due to family commitments (n=12) | “My son attends swimming and gymnastics during the week.” | |
| **Barriers to Parent STEM Involvement at Home** |  |  |
| Limited time due to competing priorities (n=22) | “Full time job, multiple kids, at home responsibilities with everyday life and homework” | |
| Limited resources and STEM materials (n=18) | “There is not enough material to use and manipulate.” | |
| Limited capability or knowledge (n=14) | “It is hard to really know what they should know by a certain age.” | |
| *Note*. Response options range from 1 to 7 (i.e., 1= Not true at all; 2= Mostly untrue; 3= Somewhat untrue; 4=  Neutral; 5= Somewhat true; 6= Mostly true; 7= Very true. Barriers items were adapted from Huijg et al., 2014. | | |

# Figure SM2

Percent Attendance Across all Treatment Conditions

*Note.* The percent attendance is calculated based on the number of actual offered workshops at each school, adjusting for workshop cancellations (e.g., due to COVID school closures).
